# SARS-CoV-2 surveillance by RT-qPCR-based pool testing of saliva swabs (lollipop method) at primary and special schools—A pilot study on feasibility and acceptability

**DOI:** 10.1371/journal.pone.0274545

**Published:** 2022-09-13

**Authors:** Anika Kästner, Petra Lücker, Martina Sombetzki, Manja Ehmke, Nicole Koslowski, Swantje Mittmann, Arne Hannich, Antje Schwarz, Kristian Meinck, Lena Schmeyers, Katrin Schmidt, Emil C. Reisinger, Wolfgang Hoffmann

**Affiliations:** 1 Institute for Community Medicine, Section Epidemiology of Health Care and Community Health, University Medicine Greifswald, Greifswald, Germany; 2 Department of Tropical Medicine and Infectious Diseases, University Medical Center Rostock, Rostock, Germany; 3 Labor MVZ Westmecklenburg, Schwerin, Germany; 4 IMD Labor Greifswald, Greifswald, Germany; Regional Medical Research Centre Bhubaneswar, INDIA

## Abstract

**Background:**

Since the onset of the COVID-19 pandemic, children have been mentally and physically burdened, particularly due to school closures, with an associated loss of learning. Therefore, efficient testing strategies with high sensitivity are necessary to keep schools open. Apart from individual rapid antigen testing, various methods have been investigated, such as PCR-based pool-testing of nasopharyngeal swabs, gargle, or saliva samples. To date, previous validation studies have found the PCR-based saliva swab pool testing method to be an effective screening method, however, the acceptability and feasibility of a widespread implementation in the school-setting among stakeholders has not been comprehensively evaluated.

**Methods:**

In this pilot study, SARS-CoV-2 saliva swab pool testing of up to 15 swabs per pool was conducted in ten primary and special schools in Mecklenburg-Western Pomerania, Germany, over a period of one month. Thereafter, parents, teachers and school principals of the participating schools as well as the participating laboratories were surveyed about the feasibility and acceptability of this method, its large-scale implementation and challenges. Data were analyzed quantitatively and qualitatively.

**Results:**

During the study period, 1,630 saliva swab pools were analyzed, of which 22 tested SARS-CoV-2 positive (1.3%). A total of *N* = 315 participants took part in the survey. Across all groups, the saliva swab pool testing method was perceived as more child-friendly (>87%), convenient (>82%), and easier (>81%) compared to rapid antigen testing by an anterior nasal swab. Over 80% of all participants favored widespread, regular use of the saliva swab method.

**Conclusion:**

In school settings in particular, a high acceptability of the test method is crucial for a successful SARS-CoV-2 surveillance strategy. All respondents clearly preferred the saliva swab method, which can be used safely without complications in children six years of age and older. Hurdles and suggestions for improvement of an area-wide implementation were outlined.

## Introduction

Since the global outbreak of the COVID-19 pandemic, efforts have been made worldwide to contain the spread of the SARS-CoV-2 virus. This has included preschool and school closures in many countries as part of the lockdown. Since the end of 2021, vaccination against the SARS-CoV-2 virus with the BioNTech/Pfizer vaccine has also been approved without restrictions in the 5- to 11-year age group and is recommended in some countries, such as the USA. The Standing Committee on Vaccination (STIKO) in Germany has limited the vaccination recommendation for children aged 5 to 11 years to those who have risk factors for severe clinical courses of COVID-19 or have relatives at high risk. After individual information, parents can also have their healthy children vaccinated. Particularly with the further spread of new virus variants, such as currently Omicron, the uncertainty about vaccination in this age group continues to increase, rendering infection surveillance one of the most important measures in pandemic containment.

Since the start of school after the summer vacations, the 7-day incidence of SARS-CoV-2 infections in Germany has increased, especially in the younger age groups among the five to 19-year-olds [1]. Hence for this age group, greater efforts are needed to contain the spread. In this context, preschool and school lockdowns must be the last measure, as studies have shown that due to the COVID-19 pandemic most children and parents experienced lockdown-related stress, the health-related quality of life of children and adolescents decreased, the prevalence of children and adolescents with mental health problems almost doubled, and their health behavior worsened [2–4]. Although it has been shown that children are less likely to be affected by severe COVID-19 courses, long-term effects such as long-COVID in this age group have not yet been investigated sufficiently [5–8]. Besides health effects of an infection, the learning loss also leads to negative long-term consequences with associated lower income in the future [9].

In addition to hygiene measures to prevent transmission of infection, testing strategies are particularly important for infection control. Regular rapid antigen testing in schools has been mandatory in Germany since April 2021. This is intended to efficiently detect infections early and thus prevent outbreaks. Rapid testing is inexpensive, provides timely results, and is therefore well suited as an area-wide surveillance strategy. Nevertheless, it should be kept in mind that antigen tests vary in sensitivity depending on the viral load compared to the gold standard of real-time reverse transcription-polymerase chain reaction (PCR) testing with the highest sensitivity [10]. For the health care system, however, regular PCR testing in the educational setting is cost-prohibitive on the one hand and resource-prohibitive on the other.

Therefore, a widely used strategy for infection surveillance is PCR-based pool testing, which has been used in the educational setting as well. The advantage of this testing strategy is the higher sensitivity of the PCR test, and the pooling of individual samples makes the procedure more cost-effective and less resource-intensive [11–14]. Pool testing is especially effective when the infection prevalence is low [15, 16]. A challenge with this method is to ensure sufficient sensitivity with the highest possible number of samples in one pool [17]. Some studies have investigated the feasibility in terms of sensitivity and efficacy of pool testing with nasopharyngeal, oropharyngeal swabs, saliva samples, and gargle samples in the school setting [18–22]. Few studies have examined the saliva swab method in this regard [23]. Only recently, a German research group investigated the implementation of the PCR-based saliva swab pool method in schools and preschools and recommended this method as a non-invasive and sensitive technique for high-throughput application and SARS-CoV-2 screening and surveillance in children in schools and preschools [24]. In another recent article, the PCR-based saliva swab pool methodology was investigated in the region of North-Rhine Westphalia, Germany by the same research group for acceptability and feasibility [25]. Thereby, an online survey was conducted among teachers who participated in the pilot project. Our study continues to build on this research taking a broader approach to investigate the acceptability and feasibility of the PCR-based saliva swab pool testing for surveillance of SARS-CoV-2 infection in schools, considering the perspectives of parents, teachers, school principals, and laboratories, using both quantitative and qualitative methods.

The aim of this study was to evaluate the feasibility of an area-wide implementation in terms of organization and acceptability of PCR-based saliva swab pool testing (lollipop method) for surveillance of SARS-CoV-2 infection in schools among stakeholders. Furthermore, possible limitations of the lollipop pool PCR method will be discussed.

## Methods

### Study design

The prospective pilot study on lollipop pool PCR testing in ten special and primary schools in Mecklenburg-Western Pomerania was conducted from 1^st^ of September until 30^th^ of September 2021. The pilot project was planned and organized by the Ministry of Education, Science and Culture, who also chose the participating schools. The focus of the special schools involved in the project was on learning disabilities. Here, students are supported who require special educational support due to a significant and prolonged impairment in learning, performance and learning behaviour at school. The evaluation of the study was carried out by the University Medical Centres Greifswald and Rostock. The study was approved by the ethics committee of the University Medicine Greifswald (BB 005/22). Both participating medical laboratories are accredited according to the DIN EN ISO 15189 standard.

### Study region

**Fig 1:**
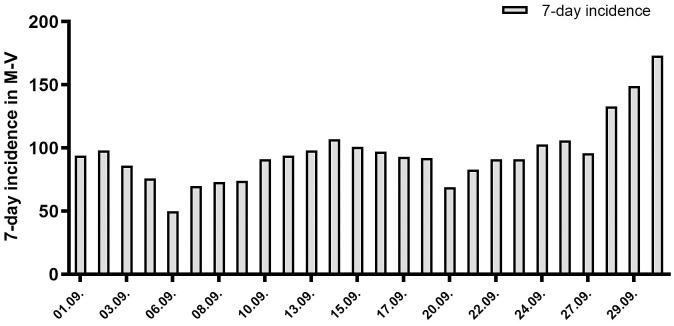
7-day incidence in Mecklenburg-Western Pomerania (M-V) during the study period. 7-day incidence in the study region in the age group of 5-14-year old children in September 2021, data base: RKI.

Mecklenburg-Western Pomerania is a rural federal state in Germany. During the period of the implementation of the pilot project, the 7-day incidence in Mecklenburg-Western Pomerania ranged between 27.4 and 44.9. According to the Robert Koch Institute (RKI), in Germany in September, 7-day incidences were highest in the 10-14-year age group (ranging between 170–227), followed by the 5-9-year age group (ranging between 142–188) [26], whereas in Mecklenburg-Western Pomerania the incidence rates in the 5-14-year age group ranged between 50.8 and 173.3 (see Fig 1).

Since April 2021, students, teachers, supporting pedagogical staff, and trainee teachers have been required to test themselves for SARS-CoV-2 virus infection twice a week using a recognized rapid antigen test as anterior nasal swab (§ 1a of the 3rd School-Corona Ordinance M-V). During the study period, those who have been fully vaccinated and those who have recovered from COVID-19 were exempted from this testing requirement.

### Conceptualization of the pilot project on lollipop pool PCR testing

Before the start of the pilot project the parents of the students were informed and had to agree to participate, otherwise the regular guidelines were applied and rapid antigen testing was performed. There were no disadvantages for children who did not participate in the project. Teachers could partake voluntarily. Teachers and school principals were informed about the procedure of the project and the sampling by the mentioned ministries before the start of the pilot project. The lollipop pool sampling was conducted under supervision of the teachers and school principals in the participating schools twice per week.

**Fig 2:**
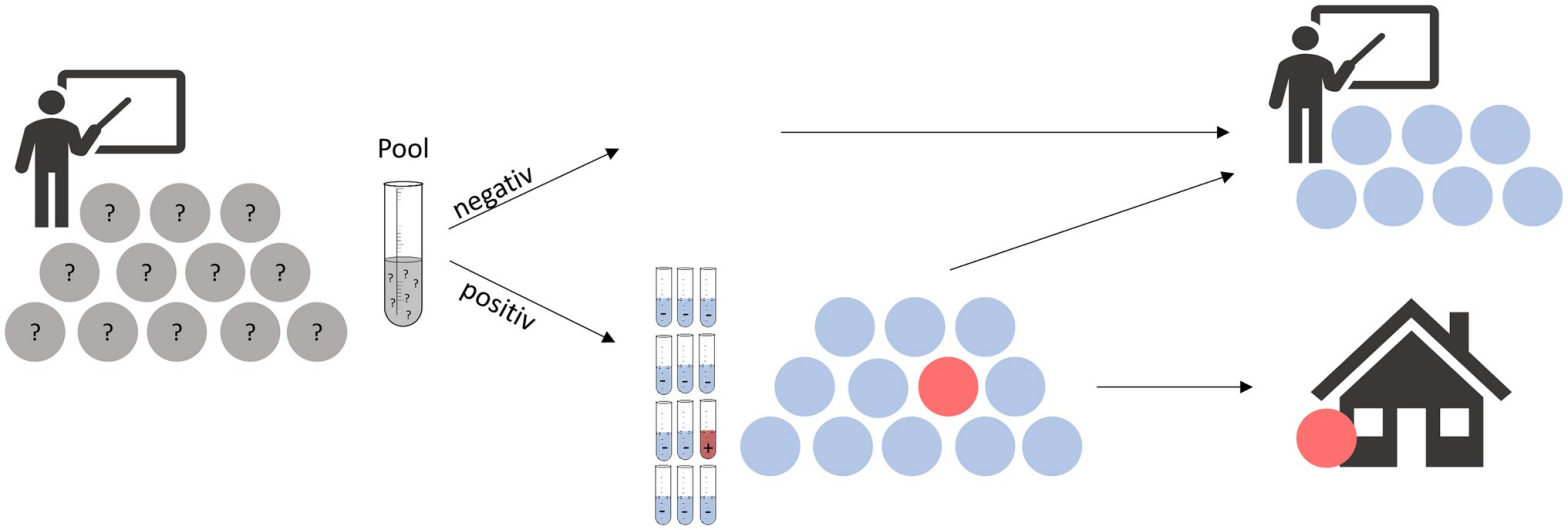
Schematic illustration of the saliva swab pool PCR testing procedure in schools. Here, the blue balls represent the SARS-CoV-2 negative individuals, whereas a red ball represents the positively tested individual(s).

Fig 2 shows schematically the procedure of the saliva swab (lollipop method) pool PCR testing. For the lollipop PCR tests, the children had to suck on the swabs for at least 30 seconds. Dry nylon throat swabs were used (Hain, Copan: flocked dry swab). With this method, it was sufficient to soak the swab with oral saliva; no throat or nasopharyngeal swab was required. Up to 15 people gave their lollipop swabs together in one empty sample tube and thereby formed a pool. No fixed number of samples per pool was specified, but rather all persons in a class who were in physical contact with each other were to form one or more pools, depending on the class size (also referred to as pooling-in-a-pod or bubble-based testing [27, 28]). The teacher recorded the names of the people who were part of one pool. The ten schools were allocated in advance to one of the two participating laboratories. The pool samples were collected from the schools by a courier.

The pooling procedure of saliva swabs is comparable to that of nasopharyngeal swabs, which has been described previously and validated in other studies [29–31]. Pool samples were transported to the laboratory in a 50mL Falcon tube. The cap of the tube was removed and the sample resuspended with 5mL guanidine hydrochloride buffer (for comparison 3mL buffer is added to an individual sample). Samples were vortexed for 3 seconds, after which laboratory personnel ensured that all swabs were completely covered by the liquid. The pool samples were then incubated for 10 minutes at room temperature. Thereafter, 2mL of the suspension was transferred to a secondary tube and RT-qPCR was performed. One laboratory examined the gene regions specific for SARS-CoV-2 ORF1 gene (open reading frame 1) and E gene (envelope) by the Cobas 6800 fully automatic PCR machine from Roche Diagnostics. The other laboratory used Seegene assays targeting the RNA-dependent RNA polymerase (RdRp), nucleocapsid (N) genes and spike (S) genes of SARS-CoV-2 in accordance with the World Health Organization (WHO) guidelines. The result of a pool sample was declared negative if no signal was detected for all genes after 40 cycles. For pool samples with at least one positive signal at RT-PCR cycle threshold (Ct) values lower than 40, the pool was resolved.

The amount of 5mL buffer solution was required to ensure that pools of up to 15 swabs were adequately covered with the solution. The 5mL solution was not reduced for smaller pools so that the dilution factor for the genetic material per swab is predictable and reproducible (dilution factor of 1.67). As shown by Christoff et al. the fixed dilution factor results in expected cycle quantification (Cq ≙ Ct) value variation constrained between 0.73 and 1.08 Ct values in pool samples compared to individual samples [31].

The results were then reported to the schools via an online tool. If the pool test result was negative, all children from the pool could attend school as usual. If a pool was positive, families were contacted by teachers (Fig 2). These students had to stay at home the next day and do an individual lollipop PCR test at home (two-stage hierarchical group testing). Therefore, parents received home testing kits prior to the start of the project.

Parents had to bring the swab to the school, where all individual swabs were collected and taken to the laboratory. The results could then be accessed by the parents via the lab’s online tool using an individual code or sample number. Students whose results were negative were allowed to return to school the next day. Students who were tested positive had to stay at home in isolation and follow the instructions of the responsible health department.

In addition to carrying out the PCR analyses of pool and individual tests, the tasks of the laboratories also included the provision of educational material (information brochures for the institutions and private households), the provision of sample material, the organisation of sample transport and the provision of test results via the labs’ own online tools.

### Evaluation of the pilot projects to lollipop pool PCR testing

The aim of the evaluation of the pilot study was to determine the feasibility and acceptability of the saliva swab PCR-based pool testing as infection surveillance strategy in schools.

According to Sekhon et al. *acceptability* is defined as multi-faceted construct that reflects the extent to which people deliver or receive a healthcare intervention and consider it to be appropriate, based on anticipated or experiential cognitive and emotional responses to the intervention [32]. Therefore, one observed behavioural measure was the drop-out rate, and self-reported measures included the evaluation of adequacy and appropriateness [32]. In addition, satisfaction with the pilot project and thus the opinion on widespread rollout were also surveyed in the context of acceptability [33].

The *feasibility* of the pilot project was evaluated with regard to the framework of the project in terms of implementation and practicability in everyday life [33]. This refers to the organization of the planned processes of the pilot project (e.g. transport of the samples to the laboratory or use of the laboratory tool to transmit results), but also to the laboratory methodology as such.

To this end, questionnaires were designed for parents, teachers, and principals to ascertain their opinions, any barriers to the implementation, and the feasibility of the lollipop pool PCR test. A second questionnaire was developed for the participating laboratories to evaluate the technical and logistical feasibility of an area-wide application on the part of the laboratories and to evaluate the number of positively tested pools and the subsequent individual tests.

The online survey was conducted from 25 September to 5 October 2021. The questionnaires were designed by the authors with support from the Ministry of Education, Science and Culture and implemented as an online survey using SoSci Survey [34]. The questionnaires consisted of corresponding questions for all groups, as well as specific questions for each group. The questions related to the initial information given/received about the pilot study, the implementation of the lollipop pool PCR tests, the notification of results and the implementation of individual tests at home. Furthermore, participants were asked whether an implementation of this test method throughout Mecklenburg-Western Pomerania was considered useful and whether the test kits should also be available from other providers (e.g. pharmacies or supermarkets).

Respondents had to consent to participate in the survey before it started. The questionnaires did not request or store any identifying or personal data. Participants were asked which school they belonged to. The identity of parents and teachers was still unknown with this information, but identification of the school principal was possible.

Regarding the questionnaires, a filter was added to the questionnaire for parents: Only if the question "Has the pool test of your child ever been positive?" was answered with "Yes", further questions about the individual testing procedure were displayed, because only in case of a positive pool test an individual retest at home was required and the child had to be quarantined until the result was available.

### Distribution of the online survey

Email invitations to complete the survey were sent to participating schools by the Ministry of Education, Science and Culture. The school principals were asked to forward the links to teachers and parents. During the study period, a reminder was sent to the school principals to forward to teachers and parents. The laboratories received their questionnaires via email from the study’s supervising scientists.

### Descriptive statistics

Questionnaires were included in the analysis if participants consented to participate in the survey and had answered at least one question. Descriptive analysis was performed using IBM SPSS Statistics (Version 28, IBM Corp, 2021). The questions were designed with single and multiple choices. If more than one answer was allowed, this was explicitly indicated. The questionnaires also offered several free text options.

Nominal variables are presented with absolute and relative numbers and metric data with median and minimum and maximum. Results were reported only for the number of people who answered the question (unless otherwise noted). The figs were created using Microsoft Excel and GraphPad Prism.

The free text fields were analyzed by content analysis following Mayring [35]. For this purpose, the quotations were paraphrased with regard to spelling and sentence order. For a unit of analysis, at least one word and at most several sentences were interpreted. Categories were formed on the basis of the generalisation.

## Results

A total of 1,630 lollipop pool tests were analysed by PCR. Of these, a total of n = 22 pools were positive with a positivity rate of 1.4% (Table 1). The number of resulting individual tests was n = 201. Overall 21 individuals were tested SARS-CoV-2 positive during the study period.

**Table 1:** Number and results of pool and individual tests per calendar week (CW).

Calendar week (CW)	Number of positive pools per performed pool tests	Positivity rate	Number of positive individual tests per performed individual test	Positivity rate
CW 35	2 / 175	1.14%	1 / 19	5.26%
CW 36	6 / 363	1.65%	4 / 49	8.16%
CW 37	4 / 366	1.09%	3 / 39	7.69%
CW 38	5 / 362	1.38%	3 / 44	6.82%
CW 39	5 / 364	1.37%	10 / 50	18.0%
**Total**	**22 / 1,630**	**1.35%**	**21 / 201**	**10.45%**

### Quantitative results of the evaluation of the pilot project

A total of 390 people started the survey. After data cleaning, N = 315 questionnaires were included in the analyses. There were n = 21 individuals who did not wish to participate in the survey, n = 53 did not answer any question and one principal filled out the questionnaire more than once and the duplicate was therefore excluded. In total, questionnaires from n = 270 parents, n = 38 teachers, and n = 7 principals were included in the descriptive analysis.

**Fig 3:**
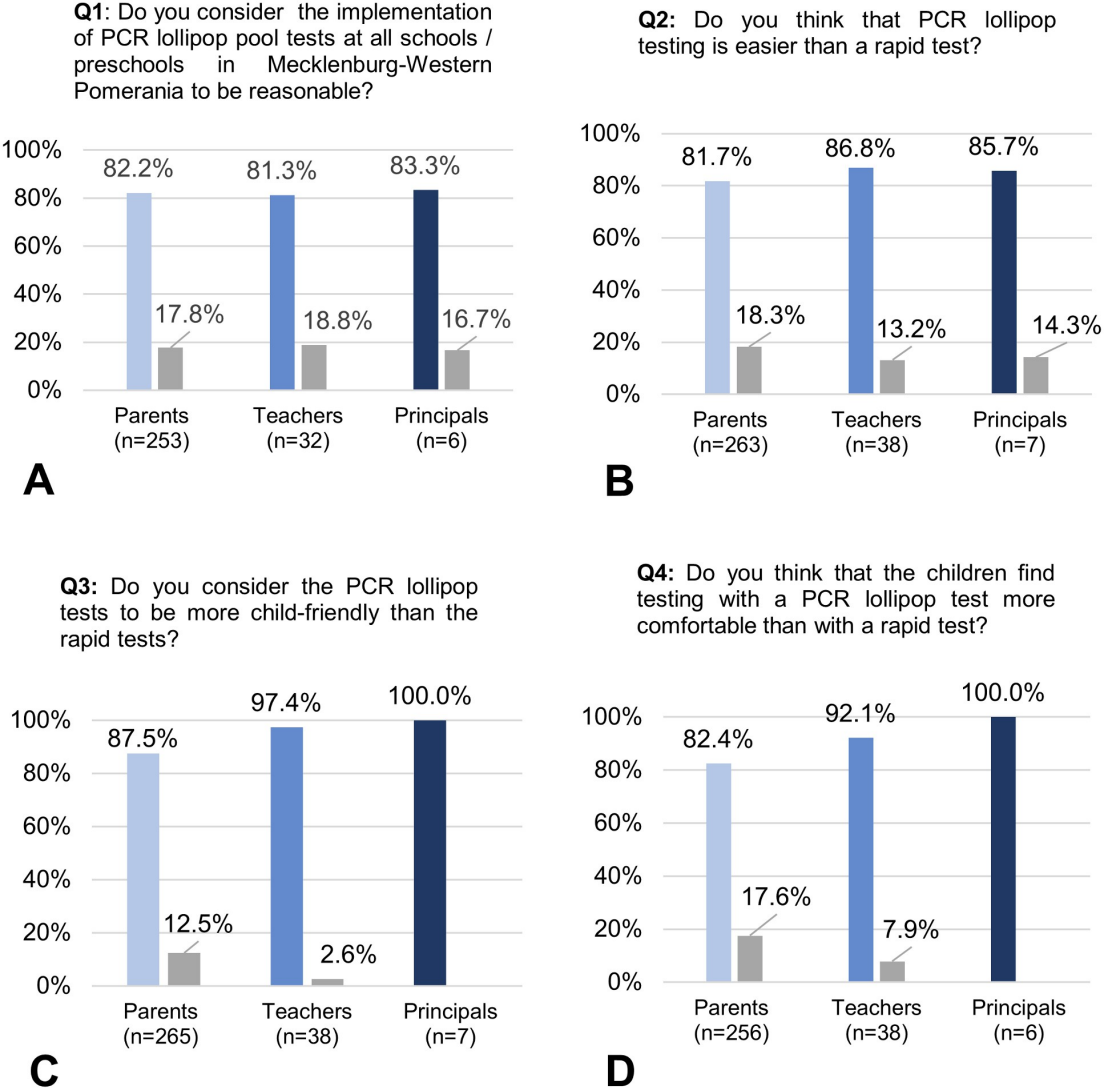
A-D. Bar graphs illustrating selected quantitative results of the pilot project on lollipop pool PCR testing. The colored bars represent the different groups and their respective agreement with the statements (light blue—parents; navy blue—teachers, dark blue—school principals). The gray bars represent the proportion of people who disagree with the statement.

The majority of parents (n = 208, 82.2%), teachers (n = 26, 81.3%) and school principals (n = 5, 83.3%) favoured using lollipop pool PCR tests in all schools in Mecklenburg-Western Pomerania over the anterior nasal rapid test (see Fig 3A). Over 80% of respondents felt that this test procedure was easier to use, over 85% felt that it was more appropriate for children, and over 80% felt that it was more pleasant for children compared to the anterior nasal rapid antigen test (see Fig 3B–3D). Further quantifiable data is shown in Table 2. Since it can be stigmatizing if a positive test result is revealed in the presence of others, parents were asked if this was an important consideration in preferring pool testing. The majority of respondents (parents: n = 171, 64.3%, teachers: n = 28, 73.7%, school principals: n = 4, 66.7%) reported that they think that it is important for the children that the test result is not known in the presence of others.

**Table 2:** Quantitative results of questionnaires from parents, teachers and school principals evaluating the lollipop pool PCR testing pilot project.

	Parents N = 270	Teachers N = 38	School principals N = 7
**Participation and Organization**			
Total number of children participating in lollipop pool testing at baseline	-	-	983
Number of children additionally enrolled during the project phase			31
Number of children dropped out during the project phase			12
How old is your child? (in years)	8 (6–15)	-	-
At your school, have teachers … / Has your child participated in lollipop pool testing?			
Yes	246 (91.8%)	7 (18.4%)	1 (14.3%)
No	22 (8.2%)	31 (81.6%)	6 (85.7%)
In your opinion, were parents … / Did you feel sufficiently informed about the lollipop pool testing process?			
Yes	241 (90.9%)	29 (78.4%)	7 (100%)
No	24 (9.1%)	8 (21.6%)	0 (0%)
Do you consider the implementation of the lollipop pool tests to be well organized?			
Yes	244 (93.5%)	23 (60.5%)	5 (71.4%)
No	17 (6.5%)	15 (39.5%)	2 (28.6%)
**Pool testing procedure**			
How many pools did you have per day? (mean, range)	-	-	18 (7–26)
How often were…. people in a pool? (percentage)	-	-	
• 1–9 persons			36.3%
• 10–14 person			61.1%
• 15 persons			1.1%
On how many days were the lollipop pool tests not collected by the laboratory at the agreed time (± 1 hour)? (mean)	-	-	1
On how many days were the individual tests collected from the laboratory at the agreed time (± 1 hour)? (mean)			2.2
On how many days were the individual tests not collected by the laboratory at the agreed time (± 1 hour)? (mean)			1.2
If you have participated in the lollipop pool tests: Has the pool test of your child … / your pool test ever been positive?			-
Yes	13 (5.2%)	1 (16.7%)	
No	236 (94.8%)	5 (83.3%)	
If the pool test of your child has ever been positive:		-	-
How often was your child’s pool positive?			
1x	8		
2x	1		
Not answered	4		
When you received the positive pool test result, did you know what to do next?			
Yes	10 (76.9%)		
No	1 (7.7%)		
Not answered	2 (15.4%)		
Were you okay with your child being quarantined initially after the positive pool test until the individual test result?			
Yes	6 (46.2%)		
No	3 (23.1%)		
Not applicable	2 (15.4%)		
Not answered	2 (15.4%)		
Has your child ever been positive in individual testing?			
Yes	2 (15.4%)		
No	8 (61.5%)		
Not answered	3 (23.1%)		
How many total days was your child quarantined due to a positive individual test?`			
0 days	1 (7.7%)		
3 days	1 (7.7%)		
5 days	1 (7.7%)		
6 days	1 (7.7%)		
14 days	4 (30.8%)		
Not answered	5 (38.5%)		
**Report of the results of the pool/individual tests**			
Has the lab’s online tool for submitting results been effective?		-	
Yes	6 (54.5%)		7 (100%)
No	2 (18.2%)		0 (0%)
I Don’t know	1 (9.1%)		0 (0%)
Not applicable	2 (18.2%)		0 (0%)
By what date were lollipop pool testing results usually available to the school at the latest? (Please indicate percentages), Mean	-	-	
Same day before 6 PM			55.6%
Same day between 6–8 PM			41.7%
Same day after 8PM			2.6%
Next day before 7AM			0%
Next day after 7AM			0.1%
Did you receive the result of the lollipop test on the same day?		-	
Yes	7 (63.6%)		5 (100%)
No	2 (18.2%)		0 (0%)
Not applicable	2 (18.2%)		0 (0%)
**Individual tests at home**			
Did difficulties arise… / Did you hear of difficulties with individual testing at home (e.g., test kit misplaced, implementation unclear, lollipop not usable)?			
Yes	1 (10.0%)	7 (21.2%)	3 (60.0%)
No	7 (70.0%)	9 (27.3%)	1 (20.0%)
Not applicable	2 (20.0%)	11 (33.3%)	1 (20.0%)
I don’t know	-	6 (18.2%)	0 (0%)
How many test kits did you give out … / receive per child?	1 (0–12)	1 (0–9)	1 (1–1)
Was the number of test kits provided (home stock) sufficient?			
Yes	169 (69.0%)	19 (55.9%)	6 (85.7%)
No, not enough	13 (5.3%)	2 (5.9%)	0 (0%)
No, too many	5 (2.0%)	1 (2.9%)	0 (0%)
I don’t know	58 (23.7%)	12 (35.3%)	1 (14.3%)
Have there been any coronavirus infections at your school this year up to the start of lollipop pool testing?	-	-	
Yes			2 (28.6%)
No			5 (71.4%)
If yes:			
How do you estimate the number of people in quarantine (per case of infection) since the lollipop pool tests to before?			
unaffected			1 (50.0%)
higher			1 (50.0%)
lower			0 (0%)

Note: Fields with a grey background indicate that the question was not asked for in the respective group

Of the 10 participating schools, 7 principals responded to the survey (Table 2). According to their answers, a total of *N* = 983 children participated during the entire study period, of which 12 participants withdrew their consent during the course of the project (drop-out rate of 1.2%). In addition, 14% of the principals indicated that teachers at their school participated in the pilot project. According to the parents surveyed, 92% of the children have participated in the project. Parents felt that the project was well organized and that they were sufficiently informed.

With regard to the lollipop pool PCR testing procedure, school principals indicated that an average of 18 pools per school were compiled per day. Here, on average, 36% of the pools consisted of 1–9 people, 61% consisted of 10–14 people, and one percent consisted of 15 people. On most days during the study period, the pool tests were picked up by the laboratories at the agreed time (on average, 7.4 of 9 days).

Of the parents surveyed, 5.2% reported (n = 13) that their child’s pool test had ever been positive. In only one case, a child had a positive pool test result twice during the pilot phase. In two cases, the individual test was positive (15.4%). In the event of a positive pool test result, 76.9% of parents knew what to do next. For 23.1% (n = 3) of parents with children with positive pool test results, it was not okay to have children stay home after a positive pool test until a negative individual test was proven. Quarantine lasted 14 days in one third of the cases. Of the participating teachers surveyed, 17% indicated that they had a positive pool-test result.

Principals’ responses regarding reporting results refer to pool test results, while parents’ responses refer to individual tests at home. All school principals received the pool test results the same day and all rated the use of the laboratory online tool for result submission as effective. On average, 56% of results were submitted to the schools the same day before 6 PM and 42% of results between 6–8 PM. In 3%, results were received the same day after 8 PM and in 0.1% the next day after 7 AM.

Parents used the online tool of the allocated laboratory to retrieve the results of the individual tests. Here, 55% of the parents rated the tool as effective, whereas 18% experienced it as not effective. The results of the individual tests were available for parents on the same day in 64% and on the following day in 18% of cases. Furthermore, 60% of principals and 21% of teachers have heard of difficulties with individual testing at home. 10% of parents have reported difficulties in administering the individual tests. Usually, for the individual tests, one test kit was provided for the parents to take home. This was sufficient according to 56% to 86% of respondents, respectively. Around 14% to 35% of respondents stated here that they did not know whether the number was sufficient. Overall, 0% to 6% of respondents said the number of received test kits was too few and 0% to 3% said the number of received test kits was too many. When asked which locations would be preferred for picking up the individual tests, 50% to 60% of respondents indicated they would like to pick them up at a pharmacy (multiple responses possible).

Of the seven school principals surveyed, 29% (n = 2) reported that they had infection cases at their school from the beginning of 2021 until the start of the pilot project. Of these two school principals, one school principal indicated that the number of individuals in quarantine was higher since the start of the pilot project compared to before, whereas one school principal indicated no change.

The questionnaire for the laboratories addressed questions on sample size and individual retesting in cases of a positive pool, logistics, and result transmission. Regarding the question whether the provision of the lollipop pool test and individual test to schools was well organized, both laboratories answered “Yes” for each test day. Furthermore, there was no day during the survey period on which the samples were not ready for collection at the schools on time or were delivered late by the courier to the laboratory. However, both laboratories reported individual problems with sample quality or sample evaluation. These were related to the sampling, missing information on the pool or individual test and thus a problematic identification of the sample.

Both laboratories were capable of transmitting the lollipop pool test results to the schools for the most part before 6 PM on the same day (lab A 76% and lab B 64%, see Table 3). Between 6 PM and 8 PM on the same day, 24% of the results were transmitted by lab A and 30% by lab B. After 8 PM of the test day, 6.5% of the results were transmitted from both labs. Only 0.14% of the results were transmitted to the schools the next day.

**Table 3:** Quantitative results of questionnaires from participating laboratories evaluating the practical feasibility of the lollipop pool PCR testing.

	Laboratory A	Laboratory B
**General questions**		
Do you consider the collection and provision of lollipop pool tests at schools to be well organized?	Yes	Yes
Do you consider the collection and provision of lollipop individual tests at schools to be well organized?	Yes	Yes
**Feasibility**		
Number of schools where the pool or individual samples were available for collection at the agreed time.	100%	100%
Number of schools whose pool or individual samples were delivered to the laboratory by the courier service at the agreed time in the above-mentioned week.	100%	100%
Were there any limitations in sample quality or problems with the evaluation?	Yes	Yes
• Sampling	Yes	Yes
• Too many tests in the pool	-	-
• Specimen storage	-	-
• Specimen transport	-	-
• Missing information for allocation of the pool	Yes	-
• Missing information for allocation of individual tests	Yes	Yes
In your opinion, is a country-wide application of the lollipop pool tests (pool and individual tests) in schools in Mecklenburg-Western Pomerania feasible with regard to laboratory capacities?	Yes	No
**Transmission of results**		
Approximately what percentage of lollipop pool PCR results were provided to schools in the time slots listed below during the survey period?		
• same day before 6 pm	76%	64%
• 6 to 8 p.m.	24%	30%
• after 8 pm	0%	6.5%
• next day before 7 a.m.	0%	0%
• after 7 a.m.	0.14%	0%
Approximately what percentage of lollipop individual PCR results were provided to parents in the time slots listed below during the survey period?		
• same day before 6 pm	43%	75%
• 6 to 8 p.m.	51%	19%
• after 8 pm	6%	0%
• next day before 7 a.m.	0%	0%
• after 7 a.m.	0%	6%
Have you found the online tool for reporting results to schools to be effective?	Yes	Yes
Have you found the online tool for reporting results to parents to be effective?	Yes	Yes

A similar pattern emerged for the individual tests. In the case of lab A, all results of the individual tests were communicated to the parents on the same day. Only 6% of the results were not provided by lab B until the next day. The labs have their own online tools for transmitting test results, and both rated their online tools as useful for transmitting results to schools (lollipop pool PCR tests) and parents (individual retest results)

### Qualitative results of the evaluation of the pilot project

In the free text fields, the respondents had the opportunity to provide further general information on the pilot project, for example on their perception of the process, implementation, problems, and provide suggestions for improvement. Specific questions were also asked, such as which further information would have been necessary for the course of the project. There were both positive and negative comments. A compilation of all categories, paraphrases and anchor examples of the statements can be found in S1 Table.

Overall, the test method was evaluated positively by parents, teachers, school principals, with only criticism regarding the organization and implementation.

Participants expressed the need or desire for improved information regarding the testing procedure and details of the swabs, such as ingredients and whether there could be late effects from sucking on them.

“*Enquiries about the material*, *active substances*, *etc*. *could not be answered competently (especially in the case of parents who reject this testing)*.”(parent, 750)

Particularly, information on how to proceed in the event of a positive pool test result needs to be enhanced, as some parents had difficulties to understand what the next steps were and how to perform the individual test. This resulted in an increased effort for teachers, as they could not only transmit the test results, but had to provide further explanations.

“*[…] in case of a positive pool*: *parents have major problems in understanding how to use the second test [*…*]*.”

(teacher, 692)

Suggestions were made on how the information could be improved. These were, e. g. information in different languages or in plain language. However, the amount of information was also considered important. Some parents might be overwhelmed by too much information.

“*[…] The parents’ letter should be reduced in size*, *as many parents are not able to extract the essential information from long texts due to their poor reading skills*.”

(teacher, 668)

In addition to information about the testing method and procedure, working parents need information regarding consequences for themselves. They worried about financial consequences if they cannot go to work, because their child has to stay at home and needs childcare and how they will prove this to their employer.

“*Who will pay for the loss of income for the day on which the second test is carried out and the child is not allowed to attend school on this day*.”

(principal, 358)

“*What should I do if my child has to stay at home*? *(Employer’s certificate)*”

(teacher, 124)

Working parents also indicated that the short-term quarantine measures led to problems organizing the next day in the event of a positive pool result. This is especially true when pool test results are communicated late in the evening.

“*[*…*] Far too late information of the result from the laboratory*. *[…]*.”

(parent, 151)

The notification of results before 6 or 7 PM worked very reliable for some of the parents and was rated positive. Others experienced difficulties.

“*[*…*] But what went very well was the communication of the results to the school principal on the homepage*.”

(parent, 450)

“*Partly late test results or transfer problems*.”

(parent, 273)

Although they liked the test method overall, teachers and school principals complained about a high workload and challenges due to the organization of tests, reporting test results, and informing parents. This took place in their free time and was also described as a mental burden.

“*[*…*] Not being able to switch off from the school day is also stressful*. *For the school principal*, *it’s a lot of pressure and organisation*. *It feels like you have more time during the day*, *but afterwards you have more work in the evening*. *Explaining to parents takes a lot of energy and nerves*.”

(teacher, 601)

Participants saw a major problem in the fact that after taking the test in the morning, children spent the entire day together in class without further protective measures. They saw this as a risk for further infections in the classroom. In this respect, the advantages of PCR-testing over rapid antigen testing were not clear to them.

“*[…] If one or more children tested positive in the pool*, *our children were with the positive children all day*, *where is the sense in that*? *There is no sense*, *because the risk of infection is much higher than in the rapid testing procedure*.”

(parent 626)

Nevertheless, some parents were convinced of the higher sensitivity of the tests and thus of an increase in safety from infections in schools.

“*I strongly advise these tests as they are more exact and detect infections well*. *[*…*] They increase safety at schools*!”

(parent, 469)

Participants of all three groups stated that the tests at schools should be uniform (lollipop pool testing vs. rapid tests), because of the potential for confusion among the students due to different testing strategies and the increased organizational burden on schools. This was a problem during the pilot study, as participation was voluntary.

The regular rapid antigen testing can also be performed at home by students. For those who did not participate in the pilot study, this option remained. Survey participants believed that lollipop pool testing, which can be done exclusively in school would eliminate the possibility of cheating and not taking the tests at all.

“*The lollipop test should be made compulsory for all students*, *as this is the only way to ensure that every student is tested for a possible infection*. *[*…*]*”

(parent, 545)

“*Everyone should be tested in school*, *I don’t think testing is always done at home*.”

(teacher 323)

The labs reported their challenges with this way of testing in schools in the free text fields. With regard to the sample quality one lab stated that some of the information on the forms was missing (date of birth, telephone number, etc.). There were difficulties when several pools of the same school were positive. In that case, a list of names of the pools was needed to ensure that positive retests followed positive pool tests. Also, it was reported that in the case of a positive pool, full retesting was not possible because some of the parents refused consent. One laboratory raised concerns about the transmission of results to households because it should be noted that digital transmission is not suitable for all persons (no technical prerequisites, language barrier, limited computer competences). Alternatively, it would have to be examined whether a transmission of the results to the schools would be justifiable under current data protection law. Interestingly, only lab A considered an extension of lollipop pool PCR testing to all schools in Mecklenburg-Western Pomerania feasible. Due to the difficulty in estimating capacity requirements in a constantly changing pandemic situation, it is difficult to assess whether an area-wide application of lollipop pool testing in schools would or would not be fully feasible considering equipment, material storing capacities and personnel limitations as well as the time factor in providing the results.

## Discussion

The aim of this pilot study was to evaluate the acceptability and feasibility of an area-wide implementation of saliva swab (lollipop) PCR pool testing for SARS-CoV-2 in primary and special schools in Mecklenburg-Western Pomerania. For this purpose, online surveys for parents, teachers, and school principals were conducted to assess the acceptability and barriers of this approach for infection surveillance. This is of particular importance since during the study period, the 7-day incidence rate was increasing significantly in this age group, vaccination rates are relatively low as the vaccination was not approved until the end of 2021 and is not recommended for healthy children aged 5–11 years in Germany, and new virus variants will potentially change transmission dynamics. In an effective infection surveillance system, SARS-CoV-2-positive individuals should be identified as soon as possible in order to prevent COVID-19 outbreaks at schools. The overall goal is to keep schools open and allow students to attend lessons in person. Requirements for an everyday infection surveillance with an area-wide application are (1) a high practicability for the schools with a regulated procedure, which does not interfere with the daily school routine, (2) tests must have a high sensitivity and specificity, and (3) testing should be effective in terms of costs and resources. Overall, our study showed that the saliva swab (lollipop) pool PCR test method in primary and special schools received great acceptance among the groups involved. However, there are some barriers with respect to feasibility, particularly if a more widespread implementation is considered.

### Acceptability of the saliva swab method (lollipop method)

The saliva swab method received great acceptance among stakeholders in the educational setting with a drop-out rate of 1.2%. Furthermore, the majority of parents, teachers and school principals considered the use of this test method to be simpler, more child-friendly and more convenient for the children compared to the anterior nasal antigen rapid test. There was a strong recommendation for the implementation of the lollipop pool PCR method in all schools in Mecklenburg-Western Pomerania. In particular, for infection surveillance in schools, a high acceptance for the testing procedure is an essential factor for long-term compliance.

To date, a few PCR-based pool testing methods, such as pooling of nasopharyngeal swabs, gargle samples, or saliva samples, are already routinely used for infection surveillance in schools. The performance of nasopharyngeal swabs is particularly stressful for younger children, as it is often associated with pain or gag reflex (depending on the sampling area). Furthermore, compared to other methods, a gargle sample, could pose a risk of choking, and anterior nasal swabs are also rather uncomfortable for children of this age group and are usually collected by an adult. Also, spitting saliva into a container already requires some dexterity, what makes it less suitable for younger children. The so-called lollipop tests have a decisive advantage here: Children only have to suck on a swab for 30 seconds, which makes the procedure comparatively more pleasant and was reflected in the high acceptance of the project, as also shown by previous studies [23, 25].

In a German study in the region of North Rhine-Westphalia by Joachim et al., the acceptance of the saliva swab method was assessed by observable measures (participation rate and drop-out rate) and was compared to oropharyngeal or buccal swabs [23]. In line with the present study, the overall drop-out rate was low (0.5%). Furthermore, the drop-out rate was significantly lower in participants who applied the saliva swab method than in participants who applied the oropharyngeal or buccal swab method (0.2% vs. 0.8%). The overall participation rate was 69.6% and did not differ significantly between swab methods (68.9% vs. 70.3%).

Recently, another German study from the North Rhine-Westphalia region by Kretschmer et al. similarly evaluated the acceptance and feasibility of a pilot project on PCR-based saliva swab pool testing and recommended its area-wide implementation [25]. Thereby, 96 teachers were asked in an online survey about the duration of sample collection, disruption of class and the overall project evaluation. The authors found that 97% of the teachers rated the overall project as outstanding and 92% rated the saliva swab pool method as more suitable for everyday use in schools compared to the individual rapid antigen tests [25]. Interestingly, in the present study, parents’ acceptability was somewhat lower than that of teachers; e.g., 97% of teachers found the PCR-based saliva swab testing more child-friendly than rapid testing, whereas this was only the case for 88% of the parents, indicating that there are to some extent differences in acceptability among those involved in the school setting. However, the proportion of parents, teachers, and principals who considered the area-wide implementation of the PCR-based saliva swab pool test method in all schools in the state to be reasonable was comparable (ranging from 81–83%).

### Feasibility of the saliva swab method (lollipop method) with regard to organizational aspects of an area-wide implementation

With regard to organizational aspects concerning a large-scale implementation, one of the main criticisms mentioned in the online surveys referred to the communication of positive pool samples to the respective parents by teachers or school principals. The results of the pools were available for the schools only and had then to be forwarded to the parents by teachers in case of a positive test result. This procedure was particularly stressful for the teachers, as they had to be on standby and call the respective parents during off-hours in the evenings. More than 40% of the pool results were not available before 6 PM. According to the laboratories, the reason for the late result transmission were the partly long distances between the schools and the laboratories. The collection of the samples was integrated into already existing courier routes, so that the samples were sometimes not collected until midday and arrived at the laboratory accordingly late. The laboratories each used their own online tool to submit results. One of these tools caused problems in the transmission of results, while the other one worked smoothly, according to the laboratory. If lollipop pool testing is to be implemented universally across the federal state, it is recommended to use a uniform online tool to communicate both pool and individual test results to parents. This approach would also relieve teachers of the task of contacting parents individually at home and from explaining further steps.

Some parents criticized the lollipop pool PCR test procedure because, unlike with the antigen test, results are not available until the evening, thus, in the meantime, the children are together all day which poses a risk of infection. In addition, classes were often divided into multiple pools, so parents could not understand why only the children whose pool was tested positive had to stay home the next day, but not their classmates. In other federal states in Germany, regulations regarding pool testing are different. In North Rhine-Westphalia, all children of a class are always assigned to one pool despite the distribution of their swabs to several tubes. As a result, an individual test must be performed for all children in one class, even if only one pool is positive [36]. Furthermore, in Bavaria and North Rhine-Westphalia, pool and individual tests are taken simultaneously [25]. Parents receive the result of the pool testing the same day before 7 PM. If a pool is positive, the individual tests of participants will be analyzed overnight. The results are available before 7 AM the next morning. Only the positively tested child or children stay home, the negative tested children–if not being a contact person—can go to school as usual [37]. Health officials can order measures as early as the day after the pool test was conducted. This procedure saves one day for negatively tested participants. For parents, this means greater planning security and less stress with their employers. Working parents can organize arrangements for possibly necessary childcare or inform their employer in the evening after receiving the positive pool result. Nevertheless, this approach does not save many resources and requires a high organizational effort for the laboratories [25]. Another option would be to perform the individual test the next day in a cohort isolation at school. Due to the higher sensitivity of the PCR pool test compared to a rapid antigen test, the infection is detected at an earlier stage when there may not yet be a risk of infection. In addition, a rapid antigen test could be performed to detect infected children with a high viral load more quickly. Performing individual tests at school would solve problems in communicating information to parents, monitoring the preanalytical setting, and simplifies the provision of test kits.

Furthermore, although parents indicated they felt well informed, there were open questions about what to do in the case of positive pools. It could be helpful to offer a website with a Q & A section, or as implemented by Kretschmer et al., the setup of a telephone hotline [25]. Care should be taken to ensure that information is adequately provided also for non-native speakers and parents with difficulties in reading.

### Feasibility of the saliva swab method (lollipop method) with regard to the laboratory method

However, in addition to a high level of acceptance and feasibility in terms of organizational aspects, other factors such as ensuring sufficient sensitivity for infection surveillance must also be considered. In general, the sensitivity and specificity of PCR detecting SARS-CoV-2 infections depends on various factors, starting with sample collection. However, the decisive factor for a standardized procedure is ultimately the viral load, whereby the probability that the PCR shows a positive result declines with decreasing viral load [38]. This aspect becomes particularly relevant when samples are pooled: A decrease in sensitivity with increasing numbers of individuals per pool is frequently reported in the literature due to a dilution effect [15, 39, 40]. It is important to note that there are different methods for sample pooling, e.g., swab pooling or pipette pooling [41, 42], with potential influence on the dilution effect of the pool sample so that the results of studies may not be directly comparable in terms of sensitivity. The applied swab pooling method (regardless of whether nasopharyngeal swab or saliva swab) has an important advantage over traditional sample pooling methods where a consistent amount of liquid is pooled per individual sample (e.g. saliva pooling): regardless of the number of samples, the dilution factor in the swab pooling method is kept constant (in this pilot study at 1.67 with 5mL buffer solution), whereas in the sample pooling method different dilution factors can emerge depending on the number of samples, as shown by Christoff et al. [31]. Therefore, the number of individuals per pool is negligible to the swab method as long as it is ensured that all swabs are covered with buffer solution (in the case of this pilot study, the maximum number was 15 swabs). The higher amount of buffer solution compared to a single sample is consistent with the approach of other studies [27, 29–31]. Accordingly, the laboratory methodology was not re-validated in this pilot study. Furthermore, it should be considered that PCR-testing is performed regularly (at least twice a week) in schools in Germany, so that in the case of individuals with an emerging infection, it can still be ensured that these can be detected promptly in the subsequent pool testing.

Furthermore, is important to note that saliva nucleic acid amplification testing has been shown to have a similar diagnostic accuracy as that of nasopharyngeal swabs nucleic acid amplification testing [43, 44]. One study found that the lollipop method detected more cases than buccal swab sampling in primary schools [23].

Nevertheless, there is an ongoing debate in the scientific community about the optimal number of samples that can be pooled in order to obtain a sufficiently high sensitivity and cost efficiency [45–47]. In a recently published study by Cohen et al., bubble-based swab samples of up to 37 swabs were pooled and tested for SARS-CoV-2 by PCR [27]. After adjusting for the dilution effect, the expected sensitivity per pool size was calculated. The authors concluded that overall, a pool size of up to 25 swabs can be recommended. In another study, up to 16 swab samples were pooled without significant differences in Ct values of the pooled sample compared to the individual sample [31]. In general, the limitation to detect samples with Ct values above 35 in a pool and thus the risk of false-negative results remains regardless of the pool method [31].

Beside the possible loss of sensitivity with an increasing number of samples in a pool depending on the pooling method, another factor plays an important role on resource and cost efficiency. The optimal number of samples per pool depends largely on the prevalence of the disease: The higher the prevalence, the higher the probability that the pool sample will be positive (which increases further the more individuals form a pool). Some studies have investigated this aspect: At a prevalence of 0.5%, the optimal number is 15 samples per pool under optimal laboratory conditions (100% sensitivity, 100% specificity) [48, 49]. However, this number decreases rapidly with increasing population prevalence. For example, at a prevalence of 1% the optimal number of samples is 11. At a prevalence of 7% the optimal number of samples per pool is 4 and at a prevalence of 13% the optimal number of samples per pool is 3. From this it can be deduced that 1.) the pool method should be continuously adjusted depending on the prevalence in the source population and 2.) with increasing prevalence, the pool method becomes ineffective both in terms of resources and costs. Considering the high acceptance of the saliva swab method, an application of saliva swab rapid testing in the school setting could be implemented at higher prevalence rates.

### Limitations of the study

One of the limitations of this study is that not all principals of the participating schools participated in the survey, so the number of children and teachers who participated, for example, differs from the actual number of participants in the study. Another limitation of this study is that due to the short study period and relatively low incidences in Mecklenburg-Western Pomerania, positive pool test results were rare. Some questions for parents could only be answered if their child had a positive pool test result, such as whether there were problems with the individual test at home or whether the result transfer by the laboratory worked well, so that the case numbers for some questions are particularly low (<10) and may be not representative. Due to the web-based survey, it cannot be ruled out that participants filled out the questionnaire more than once or incorrectly (e.g. selected the link for the wrong respondent group). An online survey also requires access to the internet, therefore not all participants may have had the opportunity to take part in the survey. Also, people who are averse to online surveys may have been less likely to participate, which may limit the representativeness. Furthermore, the saliva swab method was not re-validated in advance by the laboratories, as the primary aim of the pilot study was to evaluate the acceptability of the method and feasibility of a widespread implementation with regard to organizational aspects. Also, not all positive pool tests resulted in positive individual tests, as according to the laboratories, in some cases the entire pool could not be resolved because parents did not consent or the individual test sample was not handed in at the school. Another limitation to a large-scale implementation of the test method is the capacity of the laboratories to perform lollipop pool PCR tests. The performance of rapid antigen tests at schools for infection surveillance has so far relieved the burden on laboratories. Furthermore, with high incidences in the population, laboratories are heavily burdened with the analysis of individual PCR tests due to symptoms or positive rapid antigen tests, so that the evaluation of lollipop pool tests at schools cannot be performed with the necessary capacities on a regular basis. In addition, with a high incidence in the population, pools also become more frequently positive, leading to an increased number of necessary individual lollipop tests. Another limitation is that the selected schools may not be representative of all schools in the entire study region. Thus, only four of the eight counties of Mecklenburg-Western Pomerania were covered, of which four schools were located in the immediate surroundings of the laboratories. Most of the participating schools were located in at least small towns and thus easier to integrate into existing courier routes than more rural schools. Associated with this would be a higher logistical burden for delivery and pickup of samples to schools if the project were rolled out.

## Conclusion

In conclusion, the lollipop pool method for infection surveillance in the educational setting was highly accepted by the stakeholders compared to the anterior nasal rapid antigen test as the method is more convenient and easier for children to use. Children as young as six years of age can use the lollipop method safely without complications, which is a major advantage over the procedures regularly used in the educational setting to date. The aforementioned organizational aspects related to the reporting of pool results and the need for a common laboratory tool as well as logistical aspects were found to be hurdles to the feasibility of the pilot project which are, however, rather easy to address. Furthermore, the resource and cost effectiveness of the pool method can only be ensured in a situation with comparatively low prevalence rates. In case of increasing prevalence rates, lollipop rapid tests could be used.

Overall, our study adds to the growing body of evidence of previous implementation studies with regard to acceptability and feasibility of the PCR-based saliva swab pool testing method by a comprehensive survey of stakeholders involved in the school setting as well as laboratories using both quantitative and qualitative methods and provides recommendations for an area-wide implementation of this pilot project.

## Supporting information

S1 Table Qualitative results of questionnaires from school principals, teachers, and parents evaluating the lollipop pool PCR testing pilot project. (DOCX)
