# Supplementary material for: SARS-CoV-2 surveillance by RT-qPCR-based pool testing of saliva swabs (lollipop method) at primary and special schools—A pilot study on feasibility and acceptability
Source: PLoS One. 2022 Sep 13;17(9):e0274545. doi: 10.1371/journal.pone.0274545 (PMC9469960; doi:10.1371/journal.pone.0274545)
Supplement: S1 Table — (DOCX) [file pone.0274545.s001.docx]

**S1 Table. Qualitative results of questionnaires from school principals, teachers, and parents evaluating the lollipop pool PCR testing pilot project.**

| 1. **Wishes for additional information** | |
| --- | --- |
| Comprehensive information sheet and explanation of the procedure in case of a positive test | „[…] People without basic medical knowledge could not understand the information well in my opinion.“ (parent 402)  „How to get access to the results (foreign language parents) […].“ (teacher 124)  „Who pays the costs of the permanent testing.“ (parent 241) |
| For parents: Regulation with the employer regarding sick leave and salary | „In case of positive lollipop pool, do you get an excuse for the employer, on the day of the individual test?“ (parent 144) |
| Access and notification of test results | „Information arrives too late, must be automatized.“ (parent 637) |
| Manufacturer, ingredients, late effects | „Transparent information about the manufacturer and the harmlessness of the ingredients.“ (parent 182) |
| **2. Criticism** | |
| Children being together all day without the test result increases the risk of infection | „Test results are not known until the end of the day, children are together with possible positively tested persons the whole day.“ (teacher 150) |
| Evaluation and transfer of test results too slow/late | „Partly late test results or transfer problems.“ (parent 273) |
| Stress for teachers and school principals due to test preparation and communication of results | „[...] Not being able to switch off from the school day is also stressful. For the school principle, it’s a lot of pressure and organization. It feels like you have more time during the day, but afterwards you have more work in the evening. Explaining to parents takes a lot of energy and nerves.“ (teacher 601) |
| Competence of teachers to explain the process | „[...] I was constantly afraid these days that I would have to [...] explain to them [*the parents*] the further procedure, which I actually have no idea of myself. [...]“ (teacher 255) |
| Quarantine measures not feasible for employed parents | „[...] That all children in a pool have to be quarantined and tested again is not feasible for employed people.“ (parents 415) |
| Stress for students and parents while waiting for results | „[...] there is a constant fear of quarantine, which I consider to be very stressful for children.“ (parent 490)  „The [parents] then panicked how they should now ask the employer for time off for the following day.“ (teachers 124) |
| Class cancellations until the individual test result is available | „[...] in the worst case, many children would often have to stay away from lessons, even though they are not infected - I cannot approve [...].“ (teacher 698) |
| Lack of understanding for frequent testing of children compared to adults | „It seems disproportionate that children, who have been shown to have less critical disease courses are tested several times a week, while adults rarely have to be tested. How can it be justified that the focus is on children?“ (parents 241) |
| Test sticks not tasty and unpleasant | „The child criticizes the taste of the test stick. Quote: ‘It tastes disgusting’.“ (parents 294) |
| Misleading name for PCR lollipop test | „The name lollipop test is misleading, especially for children and misleads about the nature of the procedure.“ (parents 501) |
| **3. Positive aspects of the PCR lollipop test** | |
| Time saving | „Time-saving for all those involved [...].“ (parents 197) |
| Simple and understandable test procedure | „The procedure was quickly explained and understandable for everyone. [...]“ (teacher 75) |
| More pleasant test procedure | „It was really really great! I can only say good things. My child is much happier with it. In particular, he has inhibitions about putting the rapid test up his nose on his own, as he always reacts with heavy sneezing. Quite a great idea!“ (parents 309) |
| Provision of test kits | „There are always enough test kits packed by the school in advance in the school bag - this is working out great! [...]“ (parents 450) |
| Test administration in school | „The tests were done at school. I think that’s good.“ (teacher 516) |
| Communication of test results online or via e-mail | „[...] But what went very well was the communication of the results to the school principle on the homepage.“ (parents 450) |
| Timely notification of results | „[...] The communication of the results before 18:00 or 19:00 was very positive and should be mandatory for high acceptance of the families. [...]“ (school principal 316) |
| Higher safety | „I strongly advise these tests as they are more exact and detect infections well. [...] They increase safety at schools!“ (parents 469) |
| **4. Suggestions for optimization** | |
| Testing before school or controlled testing at home for less risk of infection and more class time | „Should be done before classes to not put further strain on children’s lesson budgets.“ (parent 612)  „It would be better to have a safe way to do it at home without people being able to cheat (just not do the test and only sign it).“ (parent 228) |
| Simultaneous individual tests in school | „It would make sense to take the individual test at the same time, so that the parents do not have to do it at home in the evening or the next morning and bring it to school in case of a positive pool result. This would also reduce the risk of errors when filling out the accompanying notes and reduce the workload of the parents in the first place. […]“ (teacher 419) |
| Smaller pool sizes for reducing the risk of false positive tests | „The group number of children tested together in one pool should be smaller, as the chance of a false positive test for the individual child then decreases. It makes me nervous.“ (parents 492) |
| Notification of the test result by another facility or payment for the work involved. | „[...] However, the additional organizational effort is clearly too high or must be compensated accordingly. Both the preparation of the class packs as well as the result query and the resulting actions are a high burden. Here, the process should be organized differently.“ (teacher 823) |
| Retrieval of results by parents through access to the online platform | „Suggestion for improvement: parents should be able to retrieve the test result themselves by accessing an appropriate website.“ (teacher 668) |
| Timely communication of results for daily planning and arrangement with employer | „In the evening, it is too late to organize childcare for the next day when it is said that the child has to be quarantined. Data must be transmitted more quickly and there must be a faster response.“ (parent 483) |
| Participation in classes with mask despite positive pool result until individual test | „[…] In addition, in the case of a positive pool result, all students of the cohort with masks should be allowed to continue attending school until the result is available (otherwise: unfair to the lollipop participants and no avoidance that the children must continue to suffer the consequences of the adults who are unwilling to vaccinate and, in principle, of the Corona pandemic).“ (parent 545) |
| Permanent implementation of the test method (at all schools) | „I think it makes a lot of sense that the tests are permanently done in the school. This way there can be no disagreements. My child finds the lollipop test much better than the previous tests. [...]“ (parent 577)  „I think that the PCR lollipop tests should also be carried out in preschools and secondary schools if the children have not been vaccinated.” (parent 606) |
| Two tests for separately living parents | „If the parents live separately, a second test kit is needed for home use.“ (parent 231) |
